# Rhino orbital mucormycosis

**DOI:** 10.11604/pamj.2021.39.114.30053

**Published:** 2021-06-09

**Authors:** Shwetambari Morghade, Rakesh Krishna Kovela

**Affiliations:** 1 Department of Neuro Physiotherapy, Ravi Nair Physiotherapy College, Datta Meghe Institute of Medical Sciences, Sawangi Meghe, Wardha, Maharashtra, India

**Keywords:** Mucormycosis, COVID-19, intracranial extension, fungal infection

## Image in medicine

We are presenting the case of a 75-year-old female who tested positive for COVID-19 in the 2^nd^ week of March with a high-resolution computed tomography (HRCT) score of 4/25 on the very next day for which she visited a hospital in sevagram where she was admitted for 7 days after which she was discharged and kept into home isolation under constant monitoring. After 2 months, on the 6^th^ of May 2021 she was brought to the hospital in Sawangi, with complaints of pain over the right side of the face which was sudden in onset, continuous, dull aching, radiating to forehead on the right side with the history of associated swelling over the right side of the face which was initially small in size and gradually increased to present size of 3 x 2 cm approx, with history of difficulty in mastication, deglutition and speech were altered, nasal stiffness over the right side since 15 days. She is a known case of hypothyroidism for 20 years, diabetes mellitus and hypertension for 10 years with chronic kidney disease. On extraoral examination patient's face was asymmetrical due to fungal infection and diffused swelling over the right side of the face and opthalmoplegia, blurring of vision, ptosis, chemosis and restricted eye movements. Intraoral examination reveals mouth opening of 25mm with diffuse gingival swelling seen in the upper right maxillary alveolar region extending anteroposteriorly from 11 to 26 regions. Magnetic resonance imaging brain and orbit reveals invasive fungal sinusitis with the cutaneous collection and intracranial extension of mucormycosis.

**Figure 1:**
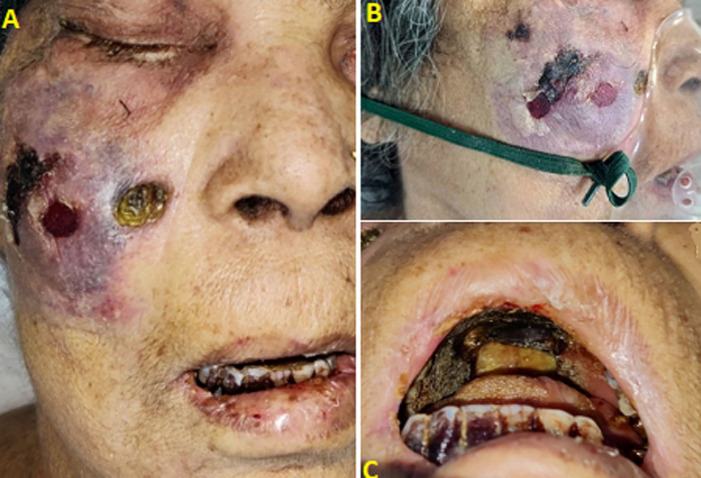
(A,B,C) rhino orbital mucormycosis

